# The complexity of premenstrual dysphoric disorder - risk factors in the population of Polish women

**DOI:** 10.1186/1477-7827-8-141

**Published:** 2010-11-14

**Authors:** Violetta Skrzypulec-Plinta, Agnieszka Drosdzol, Krzysztof Nowosielski, Ryszard Plinta

**Affiliations:** 1 Department of Womens' Disease Control and Prevention, the Medical University of Silesia, ul. Medyków 12; 40-752 Katowice, Poland; 2 Department of Physical Education and Sport, the Medical University of Silesia, ul. Medyków 12; 40-752 Katowice, Poland

## Abstract

**Background:**

Premenstrual dysphoric disorder has multiple determinants in the biological, psychological and socio-cultural domains. The aim of the study was to evaluate the risk factors for premenstrual dysphoric disorder in Polish women, considering their reproductive history, socio-economic factors, as well as lifestyle and health-related factors.

**Methods:**

2,500 females, aged 18 to 45, from the Upper Silesian region of Poland were eligible for the prospective population study. The final study sample was 1,540 individuals. The research was based on a questionnaire containing socio-economic status, general health, lifestyle, medical and reproductive history, premenstrual symptoms based on the American Psychiatric Association's criteria for diagnosing premenstrual dysphoric disorder, and patient prospective daily ratings of symptoms. The Statistica 8.0 computer software was used for statistical analysis. The value of p < 0.05 was adopted as the level of statistical significance.

**Results:**

The mean age of the studied population was 31.9 +/- 7.3 years. The majority of the studied women were married (57.9%), lived in large cities (42.0%) and had tertiary education (43.2%). The results of the study indicated that the prevalence of premenstrual dysphoric disorder was 2.1%. The final statistical analysis revealed that only tertiary education decreased the risk of premenstrual dysphoric disorder (OR = 0.08; p < 0.05).

**Conclusions:**

Our research showed that women with tertiary education are less vulnerable to premenstrual dysphoric disorder than women with a lower level of education. Reproductive and lifestyle factors seem to be play a lesser role.

## Background

Most women experience a variety of physical, behavioral and emotional symptoms during the luteal phase of the menstrual cycle. The symptoms include lethargy, persistent irritability, mood swings, changes in appetite, bloating, acne and breast tenderness [1,2]. Up to 70% of women are affected by menstrual disorders each year [2,3]. However, a definitive etiology has not yet been established [2,3]. Fluctuations in estrogen and progesterone, as well as genetic (estrogen receptor 1 variation) or neurobiological factors are believed to play an important role in premenstrual symptomatology [2-5].

Premenstrual symptoms are characterized in terms of their type, severity, and timing [6]. They may be limited to a mild discomfort or extend to the premenstrual syndrome (PMS). Depending on the degree of emotional and somatic impairment, they may even involve the most severe premenstrual dysphoric disorder (PMDD) [1,2,6,7]. The criteria for PMS and PMDD were established relatively recently (in 2000) by the American College of Obstetricians and Gynecologists (PMS) and the American Psychiatric Association (PMDD) [6,8].

PMS is a common pathology experienced by up to 50% of women of reproductive age [9,10]. By contrast, the prevalence of PMDD, i.e. where premenstrual symptoms reach a level of severity that interferes with personal, social and professional functioning, is much lower: 3% to 8% [9,10].

PMDD, most likely, has multiple determinants in the biological, psychological and socio-cultural domains. Some of the currently investigated potential risk factors for PMDD are: the quality of interpersonal relationships and cooperation, self-esteem, expectation and perception of premenstrual symptoms, stress, socio-economic factors (marital status, race, occupation and profession), biological factors (length of menses, pregnancies) and lifestyle factors (especially: dietary habits, physical exercise, stimulants and oral contraceptives) [2,10-13].

The aim of the study was to evaluate the risk factors for PMDD in Polish women, considering their reproductive history (menarche, history of previous pregnancies, deliveries, miscarriages, breastfeeding), socio-economic factors (age, place of residence, education, employment status, type of work performed, marital status), as well as lifestyle and health-related factors (physical activity, cigarette smoking, duration of cigarette smoking, number of cigarettes a day, alcohol consumption, body mass index - BMI).

## Methods

The prospective population study encompassed 2,500 women, aged 18 to 45 years old, from the Upper Silesian region of Poland. The research was conducted from January 2007 to December 2008. The inclusion criteria were: good general health, eumenorrhoea (menstruation occurring every 28 ± 4 days and lasting approximately 5 days, with a physiological loss of approximately 30-70 ml of blood), normal functions of the reproductive system (lack of major dysfunctions, such as uterine myomas, ovarian tumors), reproductive age (18-45 years old), lack of mental disorders, informed consent to participate in the study and a returned, correctly completed questionnaire. The exclusion criteria were: the presence of general systemic disorders (diabetes mellitus, hypothyroidism, seizure disorders, anemia, autoimmune disorders, endometriosis, chronic fatigue syndrome, fibromyalgia, collagen vascular disease, irritable bowel syndrome and idiopathic cyclic edema), endocrinopathies affecting the menstrual cycle and sexuality (e.g. polycystic ovary syndrome), past or present depression, a history of panic disorders, dysthymia, bipolar illness, personal disorders, use of medications affecting sexuality (antidepressants, benzodiazepines, antipsychotics), use of hormonal contraceptives and lack of sexual activity.

The statistical yearbook data was used to establish the population of females aged 18-45 living in Poland - 7,531,200. Next, the proportions of females according to age, education level and marital status in the entire population of Polish women were used to specify the study population, in order to obtain the highest representativity of the sample. The final sample size of 1,536 was calculated on the following assumptions: the population of females, aged 18-45 years living in Poland - 7,531,200, the estimated prevalence of PMDD, according to the references - approximately 3-8%, CI - 2.5%. Taking into account the relatively low response rate in epidemiological studies based on questionnaires, a population of 2,500 females was recruited. All the inclusion criteria were met by 1,540 women. The response rate was 61.6%.

The participants were recruited by the regional public opinion research center, in compliance with our specifications (quota based sampling). The eligible women were emailed or telephoned with an invitation for an interview and to participate in the study. They were subsequently asked to complete the questionnaire and the daily menstrual diary for two consecutive months.

The questionnaire was composed of four parts: 1 - socio-economic status (education level, marital status, residence, employment status, type of work performed); 2 - general health (present general systemic disorders: diabetes mellitus, hypothyroidism, seizure disorders, anemia, autoimmune disorders, endometriosis, chronic fatigue syndrome, fibromyalgia, collagen vascular disease, irritable bowel syndrome and idiopathic cyclic edema, endocrinopathies affecting the menstrual cycle and sexuality, depression, a history of panic disorders, dysthymia, bipolar illness, personality disorders, BMI), lifestyle (physical activity, cigarette smoking, alcohol consumption), medical history (medication currently used: antidepressants, benzodiazepines, antipsychotics, hormonal contraception) and reproductive history (age of menarche, duration of menstrual cycle, duration of menstruation, history of previous pregnancies, number of previous pregnancies, parity, miscarriages, breast feeding); 3 - standardized criteria for PMDD according to DSM-IV (Polish version, validated, standardized and widely used in previous studies [1]; 4 - the participants' prospective daily ratings of symptoms according to DSM-IV and the recommendations of the American Psychiatric Association (APA) (Polish version, validated, standardized and widely used in previous research) - a 4-point symptom severity scale: 0-absent, 1-mild, 2-moderate, 3-severe [8,9].

The participants were asked to fill in their menstrual diaries daily, over two consecutive menstrual cycles. Parts 1-3 were completed by each woman once, at the end of the study.

According to the American Psychiatric Association (DSM-IV), PMDD criteria require five or more of the following symptoms to present premenstrually: depressed mood or dysphoria, anxiety or tension, affective lability, irritability, decreased interest in usual activities, concentration difficulties, marked lack of energy, marked change in appetite, overeating or food cravings, hyperinsomnia or insomnia, feeling overwhelmed, other physical symptoms, e.g. breast tenderness, bloating. At least one of these symptoms must be a mood symptoms, i.e.: depressed mood or dysphoria, anxiety or tension, affective lability, or irritability. Additionally, certain phenomenal characteristics are required (present premenstrually, absent postmenstrually, causing premenstrual interference other than an exacerbation of another disorder). In our study, all of the above-specified aspects had to be confirmed by the patients' prospective daily ratings of symptoms for 2 menstrual cycles. PMDD was confirmed if: throughout the entire week prior to menstruation at least one of the four core symptoms (depressed mood, anxiety or tension, affective lability, anger or irritability) was reported as severe, and at least four additional symptoms (for a total of five) as moderate to severe, and if they were absent in the week post menses [9]. PMDD+ women constituted cases whereas healthy ones (PMDD-) were considered non-cases.

Daily alcohol consumption was defined as more than one glass of wine or one standard beer or 50 ml of alcohol.

As the number of cases (PMDD+ women) was small (N = 32), we did not perform any comparative analyses within the study population. Nevertheless, logistic regressions were used to establish potential risk factors for PMDD. The referent group, in compliance with other publications [9,10], encompassed women who: were single, not physically active, non-smokers, not consuming alcohol, aged 18-25 years old, living in rural areas, with primary/vocational education, physical workers.

The research program was approved by the Bioethical Commission of the Medical University of Silesia in Katowice, Poland.

### Statistical analysis

The results were analyzed statistically with the Statistica 8.0 computer software. Descriptive statistics were used to describe the general characteristics of the studied population. In the first stage, a univariate analysis was performed (logistic regression model) of the influence of selected factors on PMDD prevalence, i.e. reproductive (menarche, history of previous pregnancies, deliveries, miscarriages, breastfeeding), socio-economic (age, place of residence, education, employment status, type of work performed) and lifestyle (marital status, physical activity, cigarette smoking, duration of cigarette smoking, number of cigarettes a day, alcohol consumption, BMI). All the factors that were found to be statistically significant in the univariate analysis (p < 0.05) were included in the multivariate model to assess the influence of individual variables on PMDD prevalence. The value of p < 0.05 was adopted as the level of statistical significance.

## Results

### General characteristic of the study population

The mean age of the studied population was 31.9 ± 7.3 years. Most of the women were married (57.9%), lived in large cities (42.0%) and had tertiary education (43.2%). 76.0% of the women were employed, of whom 68.1% were white collar workers. Only 22.8% declared daily physical exercise. Overweight and obesity were diagnosed by BMI in 17.2% and 4.6%, respectively. Approximately one in three women (33.1%) smoked, with the average number of 15 (median) cigarettes a day and the average duration of smoking being 11.7 years. Daily alcohol consumption was declared by 9.8% of the women (Table 1).

**Table 1:** General characteristics of the study population - socio-economic and lifestyle factors

	Variable	N	%
Marital status	Single	414	27.1%
	Married	882	57.9%
	Widowed	15	1.0%
	Divorced	213	14.0%
Residence	Rural areas	414	28.0%
	Small city (below 50,000)	444	30.0%
	Large city (over 50,000)	622	42.0%
Education	Primary or vocational	377	24.8%
	Secondary	488	32.0%
	Tertiary	657	43.2%
Employment status	Employed	1141	76.0%
	Unemployed	229	15.2%
	Retired	19	1.3%
	Student	113	7.5%
Type of work performed	Manual	380	31.9%
	White-collar	813	68.1%
Physical activity		322	22.8%
Cigarette smoking		509	33.1%
Duration of cigarette smoking in years (x ± SD)		11.7 ± 6.5	
Number of cigarettes a day (median + lower/upper quartile)		15 + 10/20	
Alcohol consumption		150	9.8%
BMI (x ± SD)		22.7 ± 3.40	

BMI - Body Mass Index, SD - Standard deviation

The average menarche age in the studied population of women was 13.6 ± 1.3 years old (9 to 16). 56.5% of women had been pregnant at least once, 54.2% had delivered, 85.1% by vaginal birth (spontaneous or instrumental delivery), 17.8% by cesarean section, and 1.9% both spontaneous/instrumental delivery and by cesarean section. Spontaneous miscarriage was declared by 7.7% of the women (Table 2).

**Table 2:** Characteristics of general population - reproductive history

Variable	N	%
Menarche (years) (x ± SD)	13.6 ± 1.3	
Duration of menstrual cycle (days) (x ± SD)	28.0 ± 3.0	
Duration of menstruation (days) (x ± SD)	4.9 ± 1.1	
History of previous pregnancies	867	56.5%
Number of previous pregnancies (median + lower/upper quartile)	1 + 1/2	
Parity (median + lower/upper quartile)	0 + 0/0	
Miscarriages	115	7.7%
Breast feeding	719	58.5%

SD - Standard deviation

### PMDD prevalence and risk factors

The prevalence of PMDD in the study was 2.1% (32 out of 1,540 women). No comparative statistical analysis was performed as comparing 32 women diagnosed with PMDD with 1,508 healthy women would have been a methodological bias.

To establish the potential risk factors for PMDD, all 1,540 subjects were included in the logistic regression model. In the first stage, lifestyle, socioeconomic and reproductive variables were evaluated in a univariate logistic regression model as potential risk factors for PMDD (Table 3). The analysis revealed that divorcees, women living in large cities, unemployed, cigarette smokers and alcohol consumers were 2.68; 4.11; 2.29; 2.33 and 2.66 times more likely to suffer from PMDD compared to the referents (single, living in rural areas, employed, non-smokers, not consuming alcohol): (p values: 0.018; 0.009; 0.04; 0.018; 0.018 respectively). Women with tertiary education and white-collar workers were less likely to experience PMDD - 0.04 and 0.25 times compared to referrals (with primary/vocational education and manual workers) - p values: 0.001 and 0.0018, respectively). Neither the age of the respondents, nor reproductive factors influenced the PMDD risk (Table 3).

**Table 3:** Multiple logistic regression - socio-economic, lifestyle and reproductive risk factors for PMDD - univariate and multivariate analysis (* p < 0.05)

Variables	Univariate analysis OR (95%CI)	Multivariate analysis OR (95%CI)
Age (years)		
18-25	*Referent*	
25-30	0.62 (0.20-1.90)	-
30-35	063 (0.18-2.22)	-
35-40	1.47 (053-4.07)	-
40-45	1.04 (0.37-2.94)	-
Residence		
Rural areas	*Referent*	
Small city (population below 50,000)	0.70 (0.15-3.14)	-
Large city (population over 50,000)	4.11 (1.42-11.96)*	0.99 (0.33-2.95)
Education		
Primary/vocational	Referent	
Secondary	0.94 (045-1.93)	-
Tertiary	0.04 (0.05-0.30)*	0.08 (0.01-0.71)*
Employment status		
Employed	Referent	
Unemployed	2.29 (1.03-5.11)*	0.98 (0.01-11.80)
Retired	3.11 (0.40-24.51)	-
Student	0.50 (0.07-3.77)	-
Type of work performed		
Manual	Referent	
White-collar	0.25 (0.10-0.62)*	0.35 (0.11-1.15)
Marital status		
Single	*Referent*	
Married	1.01 (0.54-1.86)	-
Widowed	-	-
Divorced	2.68 (1.20-6.84)*	0.93 (0.25-3.43)
Physically active		
No	*Referent*	
Yes	1.03 (0.68-1.56)	-
Cigarette smoking		
No	Referent	
Yes	2.33 (1.16-4.71)*	1.95 (0.78-4.91)
Duration of cigarette smoking (years)	1.02 (1.95-1.10)	-
Number of cigarettes a day	0.10 (0.93-1.07)	-
Alcohol consumption		
No	*Referent*	
Yes	2.66 (1.13-6.27)*	2.43 (0.86-6.89)
BMI	0.97 (0.87-1.08)	-
Menarche (years)	0.87 (0.66-1.14)	-
History of previous pregnancies		
No	*Referent*	
Yes	0.77 (0.38-1.55)	-
History of previous deliveries		
No	*Referent*	
Yes	0.84 (0.42-1.69)	-
History of previous miscarriages		
No	*Referent*	
Yes	1.74 (0.60-5.08)	-
Breast feeding		
No	*Referent*	
Yes	1.12 (0.26-4.94)	-

In the second stage, a multivariate logistic regression model including the variables found to be statistically significant in the univariate analysis (divorced women, living in large cities, unemployed, cigarette smokers, alcohol consumers, tertiary education and white-collar workers) was performed to establish the risk factors for PMDD. The results showed that only tertiary education lowered the risk of PMDD (Table 3).

## Discussion

This is one of the first population-based prospective studies examining potential risk factors for PMDD. Its prospective character, the large number of respondents, as well as the strict DSM-IV criteria for PMDD diagnosis facilitated a precise estimation of PMDD prevalence in Upper Silesia and a preliminary calculation of its potential risk factors.

The prevalence of PMDD reported in literature ranges from 1.2% to 31%, depending on the methodology (retrospective, prospective, different diagnostic criteria of PMDD) [14-19]. In our study, the prevalence of PMDD was 2.1% and was much lower than in our previous retrospective studies (8.3% and 14.5%) [20,21]. However, the prospective character of this research, strict criteria for PMDD and the large study population may explain these differences.

A number of studies have shown a connection between socio-economic, biological, cultural, and lifestyle factors and PMDD. Adewuya et al. reported that older women and women with a higher level of education suffered from PMDD more frequently compare to our results [16]. Similar results were presented by Wittchen et al. [19]. However, Freeman et al. and Woods et al. found that younger age was associated with more severe symptoms of PMDD [17,22]. Cohen et al. claim that PMDD women tend to have lower education [17]. In our study, there is no correlation between age and PMDD, and a negative correlation between education and PMDD risk (women with higher education were less likely to experience PMDD). These discrepancies may be due to the different ages of the respondents in various studies (graduates in the study by Adewuya et al.; 17-24 years in the paper by Wittchen et al.), cultural differences (Nigerian study by Adewuya et al.) or lack of strict PMDD criteria in the study by Freeman et al. and Woods et al. However, our present findings are consistent with our previous study conducted on female students from Silesia, aged 18-26 [21].

The influence of other lifestyle factors on PMDD risk is not clear. Wittchen et al., Bertone-Johnson et al. and Kritz-Silverstein et al. showed that cigarette smoking was associated with a higher PMDD prevalence (OR: 3.4; 2.53 and 1.13, respectively) [19,23,24]. Alcohol consumption did not correlate with PMDD in the Wittchen et al., Kritz-Silverstein et al. and the Skrzypulec et al. studies [19,21,24]. Employment and marital status were not associated with PMDD frequency [21,25]. Similar results were obtained in our study.

Low parity, menstrual cycle length and the length of menstruation were reported to be associated with the risk for PMDD in the studies by Adewuya et al. and Kaur et al. [16,26]. This was not confirmed by other studies [13,21,25], including ours.

In some studies, women reporting significant life stress were more likely to be classified as having PMDD [2,17,22]. Studies also suggest that the majority of women with PMDD have a history of mood disorders, anxiety or personality disorders, history of sexual abuse [11,16,17,19,26], and that the use of oral contraceptives decreases the severity of PMDD symptoms [27]. These factors constituted exclusion criteria in our study.

Inevitably, there are a number of limitations to our study. Firstly, the study was based on a questionnaire and the presence of any gynecological pathologies was reported by the responders. Secondly, the influence of self-esteem, expectations about menstruation, state and trait stress level or the level of identification with traditional social roles on PMDD risk was not evaluated. The role of these factors has been reported in other studies, but not Polish. Furthermore, the prevalence of PMDD in our population was relatively small and thus the power of the logistic regression model used in the preliminary analysis of potential PMDD risk factors was low - 0.15. Finally, the homogeneity of the investigated population (women from the Upper Silesian region) does not empower us to generalize the results to the healthy female population of Poland. However, the large size of the study population, the prospective character of the research, as well as the strict criteria for PMDD diagnosis increase the value of this research.

## Conclusions

Our research showed that women with tertiary education are less vulnerable to PMDD than women with a lower level of education. Reproductive and lifestyle factors seem to play a lesser role.

## List of abbreviations

APA: American Psychiatric Association; BMI: body mass index; PMDD: premenstrual dysphoric disorder; PMS: premenstrual syndrome

## Competing interests

The authors declare that they have no competing interests.

## Authors' contributions

VSP - carried out study conception and design, data collection, statistical analysis and interpretation of data, paper review, final approval of the version to be published;

AD - carried out study conception and design, data collection, statistical analysis and interpretation of data, paper preparation, final approval of the version to be published;

KN - carried out study conception and design, data collection, statistical analysis and interpretation of data, paper preparation, final approval of the version to be published;

RP - carried out study conception and design, data collection, statistical analysis and interpretation of data, paper preparation, final approval of the version to be published;

All authors read and approved the final manuscript.
